# Anticariogenic, Antidiabetic, and Toxicology Evaluation of the Ethanolic Extract of Croton bonplandianum: An In Vitro Study

**DOI:** 10.7759/cureus.63813

**Published:** 2024-07-04

**Authors:** Vignesh Kumar G, Rajeshkumar Shanmugam, Pavithra Deenadayalan, Pradeep Manigandan

**Affiliations:** 1 Nanobiomedicine Lab, Centre for Global Health Research, Saveetha Medical College and Hospital, Saveetha Institute of Medical and Technical Sciences, Chennai, IND

**Keywords:** embryotoxicity, anticariogenic activity, cytotoxicity, antidiabetic, croton bonplandianum ethanolic extract

## Abstract

Background

Herbal medicine has gathered increasing attention in contemporary healthcare practices, offering natural remedies for a wide range of ailments such as skin diseases, liver disorders, bronchitis, and asthma. Among the plethora of medicinal plants, *Croton bonplandianum*, commonly known as "Ban Tulsi," holds significant medicinal value owing to its diverse pharmacological properties. This study investigated the cytotoxicity, embryotoxicity, antidiabetic, and anticariogenic effects of an ethanolic extract derived from *C. bonplandianum*. The research objectives were to explore the preparation of an ethanolic extract of* C. bonplandianum *and employ a multifaceted approach by evaluating its cytotoxicity, embryotoxicity, anticariogenic, and antidiabetic potentials.

Materials and methods

In this study, the β-glucosidase inhibitory and the α-amylase inhibitory assays were utilized to evaluate the antidiabetic activity of the *C. bonplandianum* ethanolic extract. The in vitro cytotoxicity activity was assessed by using the brine shrimp lethality assay (BSLA), and embryotoxicity was evaluated using zebrafish embryos and larvae. Through the agar well diffusion method and the time-kill curve analysis, the anticariogenic activity was evaluated.

Results

In α-amylase and β-glucosidase inhibitory assays, the ethanolic extract of *C. bonplandianum* showed potent antidiabetic properties, near those of standard acarbose. The cytotoxicity evaluation using the BSLA showed less toxicity. The anticariogenic activity of the ethanolic extract of *C. bonplandianum* was assessed by comparing the standard (Amoxyrite) in terms of its zone of inhibition against oral pathogens such as *Streptococcus mutans* and *Lactobacillus *species (spp.). The antibacterial efficiency was validated using a time-kill curve assay in which the study depends on the concentration of the bacterial pathogenic organisms, namely, *Lactobacillus* spp. and *S. mutans*. In embryotoxicity evaluation, there were no morphological malformations in zebrafish larvae or embryos when exposed to high concentrations of *C. bonplandianum* ethanolic extract.

Conclusion

The ethanolic extract of* C. bonplandianum *exhibited promising antidiabetic and anticariogenic effects, supporting its conventional usage in alternative medicine. The outcomes of these research analyses suggest the plant potential as a natural source of compounds with bioactive qualities and can be utilized in the healthcare and pharmaceutical industries.

## Introduction

In contemporary medicine, herbs are utilized to address the symptoms associated with chronic and acute conditions, as well as various health concerns such as cardiovascular disease, issues related to oxidative stress, inflammation, and boosting the immune system, among other conditions [1]. Herbal medicine has been widely practiced for centuries, with individuals seeking natural remedies to alleviate common ailments such as colds, allergies, upset stomachs, and toothaches. This inclination towards herbal remedies continues to grow steadily [2]. Medicinal plants play a vital role, and plant-based drugs, derived from botanical sources, represent a diverse array of compounds with pharmacological activities. These bioactive constituents, often extracted from various plant parts such as leaves, roots, bark, and flowers, possess therapeutic properties that have been harnessed for the treatment and management of various ailments [3]. Plant-derived phytochemicals are known to possess a variety of capabilities, including antipyretic, antibacterial, antioxidative, and anticariogenic effects [4].

*Croton bonplandianum *is commonly referred to as "Ban Tulsi" due to its similarity to the flower and leaves of the cymes of Tulsi. It is also a monoecious exotic weed and belongs to the family Euphorbiaceae [5]. This plant species holds considerable medicinal significance, particularly in the regions where it is found [6]. One notable application of *C. bonplandianum* is the utilization of its stem latex for medicinal purposes, predominantly in treating wounds and fresh cuts to arrest bleeding [7]. Moreover, research indicates its efficacy in combating microbial infections, making it a valuable resource for managing skin ailments, cuts, and wounds [8]. There are many phorbol esters and diterpenes in *C. bonplandianum *seeds, including 12-ortho-tetradecanoylphorbol-13-acetate (TPA) and myristoyl phorbol acetate (MPA). TPA, a known carcinogen, has been found to impact prostaglandin metabolism, and the extracts from this plant exhibit antimicrobial and antitumor activities [9]. *C. bonplandianum* demonstrated notable β-glucosidase and α-amylase inhibitory activity, surpassing that of acarbose. Additionally, it is purported to possess antiseptic properties, further enhancing its therapeutic potential [10]. Despite this, there have been limited studies on the *C. bonplandianum* ethanolic extract. This research concentrated on investigating the potentiality of the ethanolic extract of *C. bonplandianum* for its cytotoxic effects, anticariogenic properties, and antidiabetic properties and finally evaluating its embryotoxicity.

The objective of this study is to prepare an ethanolic extract from *C. bonplandianum* and evaluate its cytotoxic effect using the brine shrimp lethality assay (BSLA) and embryotoxicity assay with zebrafish larvae, determining their viability and hatching rate. Additionally, the antidiabetic potential will be investigated through β-glucosidase and α-amylase enzyme assays at different concentrations. Furthermore, the extract's anticariogenic properties will be assessed using agar well diffusion techniques against oral pathogens such as *Streptococcus mutans* and *Lactobacillus *spp., along with time-kill curve analysis. The findings obtained from these assessments will have significant inferences for scientific applications in the field of health-related categories, emphasizing the importance of utilizing the curative benefits of medicinal herbal plants [11,12].

## Materials and methods

Plant extract preparation

To prepare a plant extract, 15 grams of leaves were taken and thoroughly cleansed well with water to eliminate any traces of dust particles. The leaves were crushed using mortar and pestle. Subsequently, the crushed leaves and a volume of 25 mL of ethanol were mixed. Subsequently, the ethanolic mixture underwent ultrasonic treatment using a digital sonicator for 30 minutes. Following the sonication process, the ethanolic mixture of leaves was moved to an orbital shaker and agitated for 30 minutes [13]. Afterwards, the ethanolic leaf extract of *C. bonplandianum* was obtained after filtration and centrifugation. The ethanolic extract of 22 mL obtained after filtration was subsequently used for future study purposes. 

Cytotoxic effect 

BSLA

For the preparation of the solution, a quantity of 2 g of salt was measured and subsequently dispersed in 200 mL of deionized water. The experiment utilized six-well plates, with each well containing 10-12 mL of saline water. Following this, a total of 10 nauplii were gradually introduced into each well, while each well was enriched with varying concentrations of the ethanolic extract derived from *C. bonplandianum*. The plates were then incubated at ambient temperature for 24 hours. Following 24 hours, the plates were meticulously examined and counted to determine the number of viable nauplii present, and that number was then calculated using the percentage of live nauplii [13].

In vitro antidiabetic activity 

The procedure followed a protocol that was modified from previous research of Shanmugam et al. method [14]. The in vitro antidiabetic activity of *C. bonplandianum* ethanolic extract involved two methods: β-glucosidase and α-amylase enzyme inhibitory assays.

α-Amylase Assay

The analysis of maltose release was conducted in the α-amylase inhibitory test to assess the inhibitory effect of α-amylase. The *C. bonplandianum* ethanolic extract was pre-incubated with a 100 µg/mL solution of α-amylase for 30 minutes at a temperature of 37°C. The concentration ranged from 10 to 50 µg/mL. An aqueous solution containing starch at a concentration of 100 µg/mL (1% w/v) was introduced, and an incubation period of 30 minutes was conducted at a temperature of 37°C. Subsequently, a 96 mM solution of 3,5-dinitrosalicylic acid (DNSA) was added. After the cessation of the reaction, the solution underwent further analysis. The control group consisted of sodium phosphate buffer. An enzyme-linked immunosorbent assay (ELISA) plate reader was used to observe the absorbance at a wavelength of 540 nm. As a positive control, acarbose was used, and the study was carried out in triplicate. The estimation of the percentage inhibition of α-amylase was performed.

β-Glucosidase Assay

In the assay evaluating the inhibitory activity of the β-glucosidase enzyme, an ethanolic extract derived from *C. bonplandianum* was tested at concentrations ranging from 10 to 50 µg/mL. In the presence of a 0.2 M Tris buffer adjusted to pH 8.0, the ethanolic extract was mixed with a starch solution (2% w/v maltose or sucrose). Following a five-minute incubation period at 37°C, 1 mL of β-glucosidase enzyme (1 U/mL) was added, and the reaction was allowed to proceed for 40 minutes at room temperature. Termination of the reaction was achieved by adding 2 mL of 6 N HCl. The positive control utilized in this analysis was acarbose. The percentage of β-glucosidase inhibition was determined by measuring the absorbance at 540 nm.

Anticariogenic activity

The potential anticariogenic properties of the ethanolic extract from *C. bonplandianum* were evaluated against oral pathogens including *Lactobacillus *spp. and *S. mutans *[15]. The activity was assessed using Mueller Hinton agar as the medium to determine the zone of inhibition. Mueller Hinton agar was prepared and subjected to sterilization. Subsequently, the sterilized plates were filled with the media and left to solidify. Wells were then created using a suitable cutter, and the test organisms were swabbed onto the agar surface. Various concentrations of the *C. bonplandianum* ethanolic extract were loaded into the wells, and the plates were subsequently incubated at 37°C for 24 hours. After the incubation period, the measurement of the zone of inhibition surrounding each well was measured to assess the extract's activity against the tested pathogens.

Time-kill curve analysis

A 1-millilitre volume of the bacterial suspension containing *S. mutans* and *Lactobacillus *spp.was added to 9 mL of Mueller Hinton broth [12]. An ethanolic extract of *C. bonplandianum *was added to the Mueller Hinton broth at concentrations of 25 µg/mL, 50 µg/mL, and 100 µg/mL. The final microbial concentration was 106 colony-forming units per millilitre of microorganisms. After that, the mixture was incubated for varying intervals of time (zero, one, two, three, four, and five hours) at 37°C with agitation at a speed of 200 rpm. At regular intervals, a 600 nm wavelength is used to calculate the percentage of perished cells.

Zebrafish embryo collection and treatment

Maintenance of Zebrafish and Exposure to Ethanolic Extract

A breeding tank was established to acquire zebrafish embryos, consisting of five female and seven male zebrafish. Following successful mating, viable eggs were gathered and meticulously washed, ensuring that they were washed a minimum of three times using freshly prepared E3 media. Subsequently, the fertilized eggs were then distributed into six-well plates, with each well containing 2 mL of fluid. The study involved the establishment of three sets of experimental and control groups [16]. In the conducted experiment, the E3 media was augmented with different concentrations of a recently generated stock solution containing an ethanolic extract of *C. bonplandianum*. The solution was subjected to sonication for 15 minutes to achieve uniform dispersion while simultaneously maintaining a pH range of 7.2-7.3. A range of concentrations from 5 to 80 µg/mL were administered to healthy fertilized embryos for a duration of 24-96 hours post-fertilization. To prevent interference, deceased embryos from the groups treated with *C. bonplandianum* ethanolic extract were removed every 12 hours. In order to mitigate the impact of light interference, a layer of foil was covered on all experimental plates, which were kept at a temperature of 28°C.

Evaluation of Zebrafish Embryos

The development of zebrafish embryos was carefully monitored after fertilization using a stereo microscope. From 24 to 78 hours post-fertilization, the embryos were exposed to various concentrations of *C. bonplandianum* ethanolic extract, ranging from 5 to 80 µg/mL, including 5, 10, 20, 40, and 80 µg/mL. The study focused on assessing embryonic mortality, hatching rates, and viability rates which were measured at 24-hour intervals. Any abnormalities observed among the embryos and larvae in both the control and treatment groups were recorded using a Coslab model HL-10A light microscope (Ambala Cantt, India). Images of any malformed embryos were captured, and the percentage of abnormal embryos was noted every 24 hours.

Statistical analysis

Every experiment was carried out in triplicate. In the graphical representation, the standard error of the mean (mean ± SEM) analysis is provided for the error bar, and Welch's correction test was used to identify the significance of the P-value expressed as <0.05 by using the GraphPad Prism software. 

## Results

BSLA

**Figure 1:**
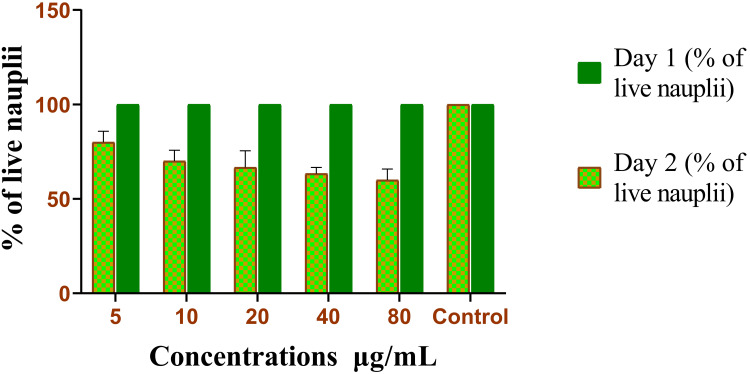
Cytotoxicity activity of C. bonplandianum ethanolic extract using BSLA The values are provided in triplicates, and the error bars present in a graph of the BSLA represent the SEM. The statistical test used was Welch's correction. A value of P≤0.05 was considered statistically significant. *C. bonplandianum*: *Croton bonplandianum*; BSLA: brine shrimp lethality assay; SEM: standard error of the mean

The cytotoxic effects of the ethanolic extract of *C. bonplandianum* were assessed through the utilization of the brine shrimp lethality experiment, as depicted in Figure 1. This analysis is frequently used to evaluate the cytotoxicity of substances by measuring their impact on the survival of brine shrimp nauplii. A drug-free control group was kept in the present study to indicate the percentage of live nauplii. The cytotoxicity assessment results showed that the *C. bonplandianum* ethanolic extract exhibited different outcomes on nauplii existence at different concentrations. At 5 μg/mL of the *C. bonplandianum* ethanolic extract, around 80% of the nauplii survived. However, when the concentration was increased to 80 μg/mL, only 60% of the nauplii survived.

In vitro antidiabetic activity

**Figure 2:**
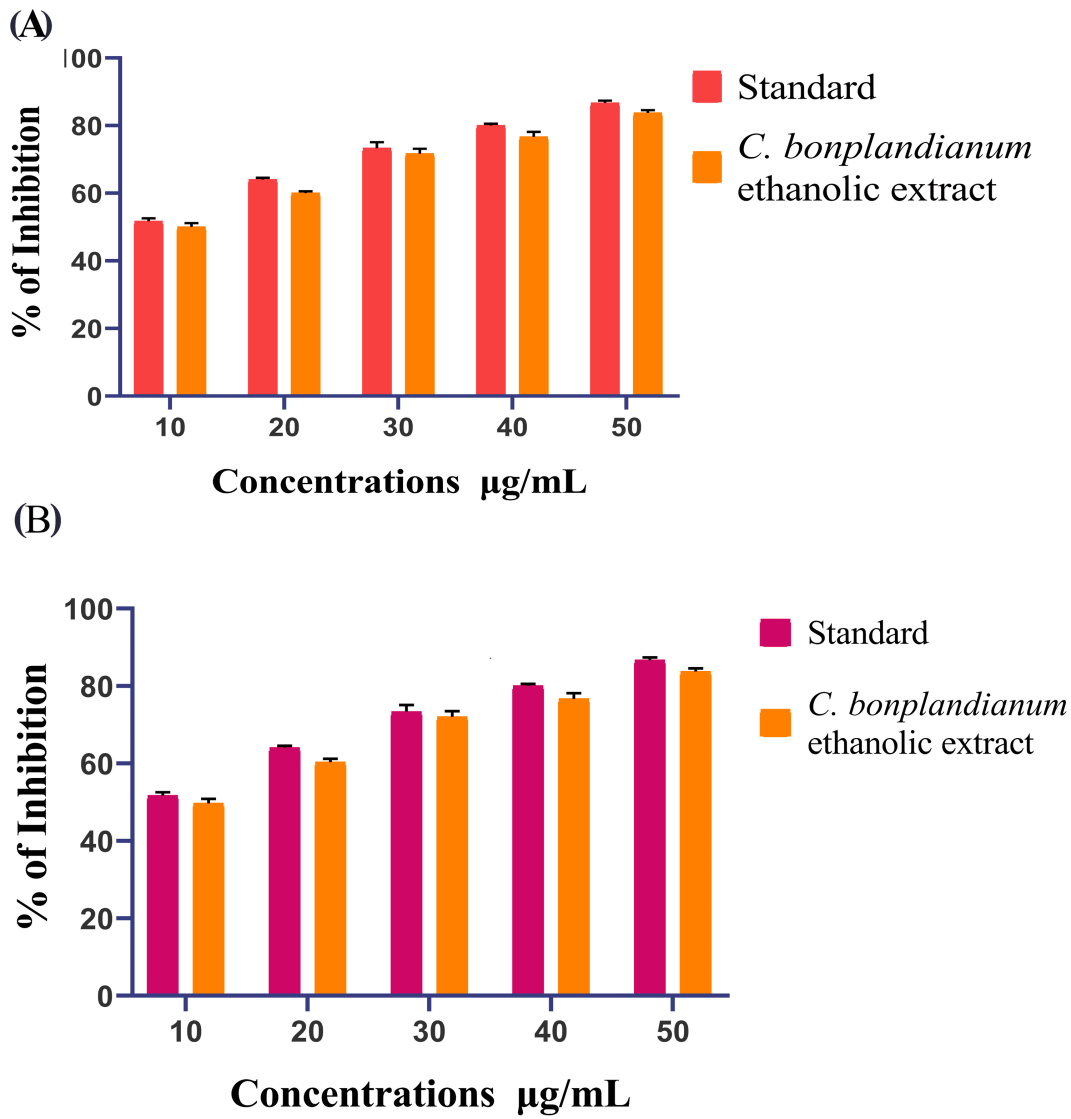
In vitro antidiabetic activity of C. bonplandianum ethanolic extract: (A) α-amylase inhibitory assay and (B) β-glucosidase inhibitory assay The results of the α-amylase inhibitory assay and β-glucosidase inhibitory assay are given in triplicate. The error bars on the bar graph represent the SEM. A Welch's correction statistical test was conducted to assess the significance. A value of P≤0.05 was deemed to have statistical significance. *C. bonplandianum*: *Croton bonplandianum*; SEM: standard error of the mean

The research examining the antidiabetic properties of the ethanolic extract of *C. bonplandianum* revealed notable inhibitory effects on the activities of β-glucosidase and α-amylase, as illustrated in the following figures (Figure 2A, 2B). The observed decrease in enzymatic activity, which is dependent on the concentration, indicates a possible mechanism for regulating glycemic control. The α-amylase inhibition by the ethanolic extract of *C. bonplandianum *suggests a decrease in the breaking down of complex carbohydrates, resulting in a reduction in glucose synthesis. The standard demonstrates an α-amylase activity of 88% at a dose of 50 μg/mL, while the ethanolic extract of *C. bonplandianum* exhibits a higher activity of 85%. At the minimum dose of 10 μg/mL, the ethanolic extract of *C. bonplandianum* demonstrates an inhibition percentage of 45%, while the standard extract exhibits a percentage inhibition of 52% (Figure 2A). The concurrent detection of β-glucosidase inhibition implies a potential disparity in glucose uptake inside the small intestine. Applying acarbose as a standard in comparison studies provides important information about the ability of the *C. bonplandianum* ethanolic extract to regulate glucose absorption and carbohydrate metabolism. In the β-glucosidase inhibitory assay, the ethanolic extract of *C. bonplandianum* showed inhibition percentages ranging from 85% to 88%, at a normal dosage of 50 μg/mL. Nevertheless, at the lowest concentration of 10 μg/mL, the standard percentage of inhibition was observed to be 52%, while the ethanolic extract of *C. bonplandianum* demonstrated a percentage of inhibition of 49% (Figure 2B). The combined significance of these effects suggests that the ethanolic extract of *C. bonplandianum* may play a significant role in reducing postprandial hyperglycemia. This emphasizes the potential of this extract for further research in the field of antidiabetic treatments.

Anticariogenic activity

**Figure 3:**
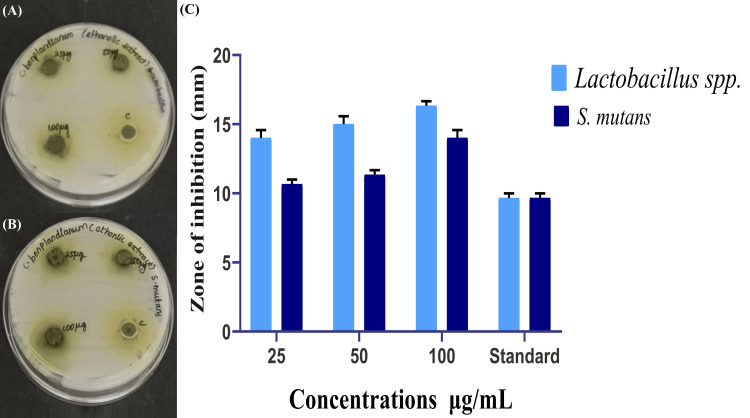
Anticariogenic activity of C. bonplandianum ethanolic extract: (A) Lactobacillus spp., (B) S. mutans, and (C) graphs showing the zone of inhibition The values for the zone of inhibition in the agar well diffusion method are provided in triplicate, and the error bars present in the graph indicate the SEM. A Welch's correction statistical test was performed to evaluate the significance. P≤0.05 was statistically significant. *C. bonplandianum*: *Croton bonplandianum*; *Lactobacillus* spp.: *Lactobacillus* species; *S. mutans*: *Streptococcus mutans*; SEM: standard error of the mean

The agar well diffusion method was employed to assess the antibacterial efficacy of the ethanolic extract derived from *C. bonplandianum *(Figure 3A, 3B). The resulting zone of inhibition was graphically depicted in Figure 3C. The ethanolic extract exhibited a zone of inhibition measuring 17 mm when subjected to loading and testing against* Lactobacillus *spp. at a dose of 100 µg/mL (Figure 3A). In comparison, the standard exhibited a smaller zone of inhibition measuring 9 mm. Similarly, when tested against *S. mutans*, the ethanolic extract of *C. bonplandianum* displayed a 14 mm zone of inhibition at the same concentration of 100 µg/mL, whereas the standard demonstrated a 10 mm zone of inhibition (Figure 3B). Notably, consistent levels of inhibition were observed for *Lactobacillus *spp. across three distinct concentrations. In comparison to the commercial antibiotic Amoxyrite, the ethanolic extract of *C. bonplandianum* demonstrated a higher zone of inhibition of 17 mm against* Lactobacillus *spp.at a concentration of 100 µg/mL.

Time-kill curve analysis

**Figure 4:**
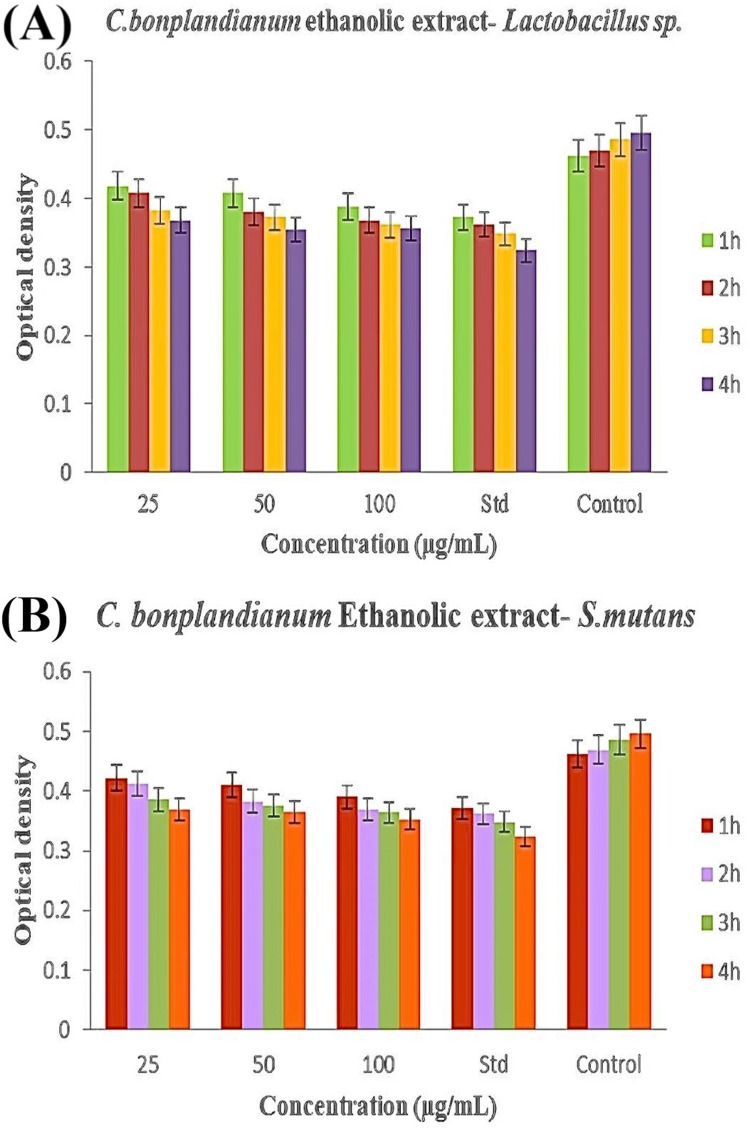
Time-kill curve assay of C. bonplandianum ethanolic extract: (A) Lactobacillus spp. and (B) S. mutans The SEM is shown by the error bars in a graph of the time-kill curve assay. The values are in triplicate. Welch's correction was the statistical test that was used. A significance level of P≤0.05 was thought to be statistically significant. *C. bonplandianum*: *Croton bonplandianum*; *Lactobacillus* spp: *Lactobacillus* species; *S. mutans*: *Streptococcus mutans*; SEM: standard error of the mean

The time-kill curve assay revealed that the antibacterial activity of the ethanolic extract derived from *C. bonplandianum* exhibited a concentration-dependent relationship in comparison to the control group. Across all concentrations tested (25 µg/mL, 50 µg/mL, and 100 µg/mL), there was a significant reduction in both *S. mutans* and *Lactobacillus *spp. counts. Notably, at the highest concentration of 100 µg/mL, there was a noticeable decline in *S. mutans *colonies at the fourth hour, indicating rapid bactericidal efficacy (Figure 4B). Interestingly, while *S. mutans* exhibited moderate susceptibility, *Lactobacillus *spp.showed an increased antibacterial effect at the same fourth hour (Figure 4A, 4B). The *C. bonplandianum* ethanolic extract led to a significant reduction in *Lactobacillus *spp. counts at all concentrations compared to the control group, with a particularly prominent decrease at the highest concentration (Figure 4A). These results underline the concentration-dependent antibacterial properties of the *C. bonplandianum* ethanolic extract against the tested bacterial strains.

Embryotoxicity evaluation

**Figure 5:**
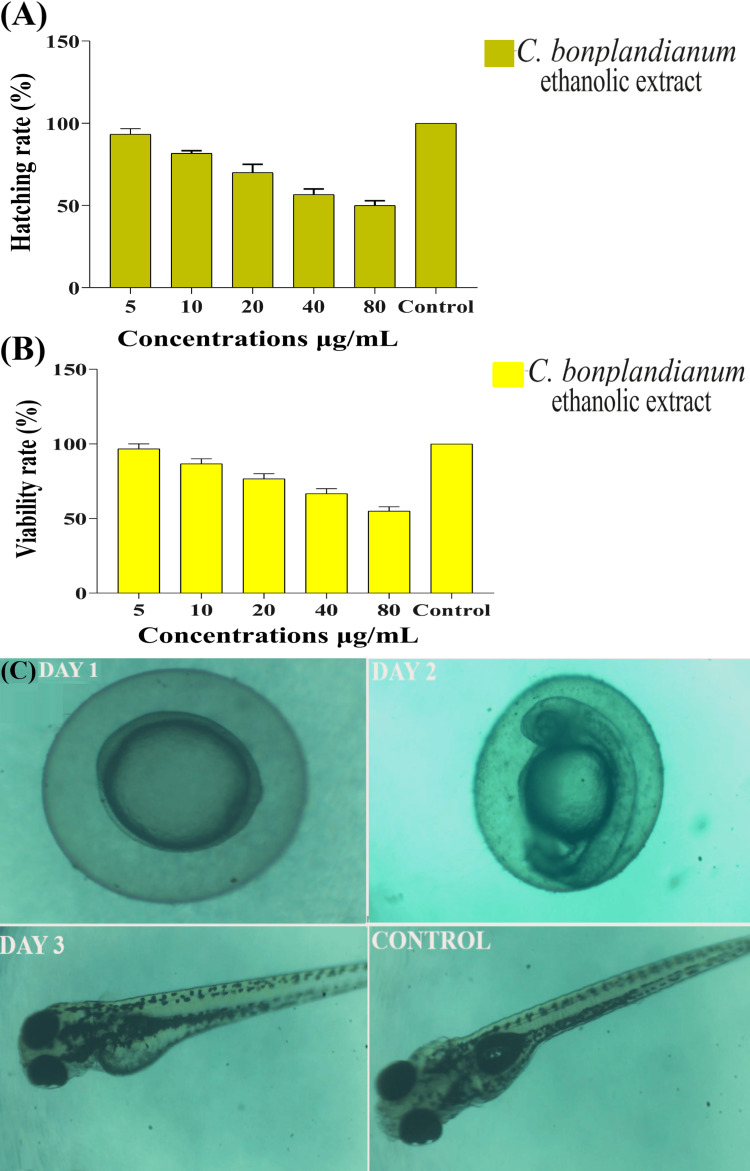
The embryotoxicity evaluation of C. bonplandianum ethanolic extract on zebrafish embryo: (A) hatching rate, (B) viability rate, and (C) morphological image of embryos Figure 5C represents the zebrafish embryonic toxicology imaging. DAY 1 shows healthy embryos. DAY 2 shows a fully formed embryo. DAY 3 shows hatched zebrafish larvae. There are no morphological deformities, such as bent tail or spine, and pulmonary edema. CONTROL shows normal zebrafish larvae for comparative analysis. The values of the hatching rate and viability rate are provided in triplicate. The SEM is shown by the error bars in a graph. Welch's correction test was used, and the value of P≤0.05 was statistically significant. *C. bonplandianum*: *Croton bonplandianum*; SEM: standard error of the mean

It has been demonstrated that the hatching rate of embryos was 55% at 80 µg/mL of *C. bonplandianum* ethanolic extract, 60% at 40 µg/mL, 75% at 20 µg/mL, 85% at 10 µg/mL, and 90% at 5 µg/mL (Figure 5A). By comparison, the hatching rate of the blank control was 100% (Figure 5A). Zebrafish embryo viability was determined to be 50% at 80 µg/mL of *C. bonplandianum* ethanolic extract concentrations, 70% at 40 µg/mL, 80% at 20 µg/mL, 90% at 10 µg/mL, and 100% at 5 µg/mL (Figure 5B). In contrast, the blank control exhibited a viability rate of 100% for the embryos (Figure 5B). The morphological imaging revealed the presence of partially developed viable embryos within the egg on the first day, the presence of fully developed viable embryos within the egg on the second day, and the emergence of healthy zebrafish on the third day (Figure 5C). No somatic malformations, such as a bent tail and bent spine, were observed, and there was no indication of edema.

## Discussion

The current study focused on examining the cytotoxic, antidiabetic, and anticariogenic properties of the ethanolic extract derived from *C. bonplandianum*. The results indicate potential bioactivities that justify further research [17]. The results of the BSLA demonstrated that the extract had a cytotoxic impact that depended on the concentration of the ethanolic extract derived from *C. bonplandianum*. Lower concentrations had a negligible effect on the survival of nauplii [18], but greater concentrations showed a steady decline in viability. The findings of this study suggest the presence of a cytotoxic impact and show how effective *C. bonplandianum* is against brine shrimp.

The inhibitory effects of the ethanolic extract on α-amylase and β-glucosidase enzymes were observed, indicating a potential mechanism for the regulation of blood glucose levels [19]. The decrease in enzyme activity, which is dependent on the concentration, suggests that the extract may impede the breaking of carbohydrates and the absorption of glucose [20]. In comparison to the standard, the extract demonstrated inhibitory activity that was either comparable or somewhat lower. This suggests the need for further studies to enhance its effectiveness and investigate potential synergistic effects when used in conjunction with existing antidiabetic medications [21]. The results of the agar well diffusion assay demonstrated the extract's potential antibacterial action against *Lactobacillus *spp. and *S. mutans*. In several cases, the extract exhibited zones of inhibition that surpassed those observed with the standard antibiotic. The concentration-dependent bactericidal activity against both strains was validated by the time-kill curve assay, with *Lactobacillus *spp. demonstrating higher susceptibility [22]. These findings indicate that the extract has the potential to be used as a natural medicinal agent for improving oral health, namely, against germs that cause tooth decay. More studies are necessary to clarify the precise bioactive components accountable for the antibacterial activity and evaluate their effectiveness in living organisms. The hatching and survivability rates of zebrafish embryos that were subjected to the ethanolic extract of *C. bonplandianum* were found to be promising when compared to the previous studies [23]. Even at the maximum quantity examined (80 µg/mL), a substantial proportion of embryos successfully hatched and maintained their viability. The lack of morphological abnormalities indicates that the extract demonstrates low levels of embryotoxicity in the quantities that were evaluated. This research presents initial findings that provide support for the possible therapeutic uses of the ethanolic extract derived from *C. bonplandianum*. Further investigation is required to clarify the fundamental processes of action, enhance their effectiveness, and guarantee their safety for human application.

Limitations

This study investigates the therapeutic potential of the ethanolic extract of *C. bonplandianum* while also recognizing particular limitations. The study lacks the identification of specific bioactive compounds, which is essential for analyzing their mechanisms. Furthermore, the study heavily depends on in vitro experiments, which must be confirmed through in vivo experiments to properly evaluate effectiveness and safety. The limiting focus on the ethanolic extract of *C. bonplandianum* limits broader comparisons, and the varying responses observed at different concentrations require a more thorough investigation of the dose-response affinity. Furthermore, a limited evaluation of antimicrobial effectiveness against only two bacterial strains (*Lactobacillus *spp. and *S. mutans*) limits the applicability of the results to a broader range of applications. Even though the extract's therapeutic uses offer valuable insights, it is essential to acknowledge and overcome these limitations in future research to improve our understanding.

## Conclusions

The research results collectively highlight the diverse pharmacological effects of the ethanolic extract of *C. bonplandianum*. The extract revealed significant antidiabetic, anticariogenic, and antibacterial properties, showing comparative or even greater effectiveness compared to conventional chemicals in specific instances. These findings support the traditional utilization of *C. bonplandianum *ethanolic extract in alternative medicine and provide a scientific basis for its therapeutic potential. Further investigation is required to separate and determine the exact bioactive compounds accountable for these effects. Furthermore, it is essential to carry out in vivo experiments to confirm and improve upon these beneficial results. The ethanolic *C. bonplandianum* extract exhibits potential as a natural repository of bioactive compounds that could be used in modern healthcare practices, emphasizing the importance of integrating medicinal plants into mainstream medicinal approaches.
